# Species Classification and Quality Assessment of Cangzhu (Atractylodis Rhizoma) by High-Performance Liquid Chromatography and Chemometric Methods

**DOI:** 10.1155/2013/497532

**Published:** 2013-07-25

**Authors:** Yong-Gang Xia, Bing-You Yang, Qiu-Hong Wang, Jun Liang, Di Wang, Hai-Xue Kuang

**Affiliations:** Key Laboratory of Chinese Materia Medica, Heilongjiang University of Chinese Medicine, Ministry of Education, Harbin 150040, China

## Abstract

Fast and sensitive high-performance liquid chromatography (HPLC) coupled with chemometric methods was utilized to assist in the quality assessment of Cangzhu (Atractylodis Rhizoma). By comparative analysis of chromatographic profiles, twelve common peaks were selected for multivariate analysis. Principal component analysis (PCA) and orthogonal partial least squares discriminant analysis (OPLS-DA) of the chromatographic data demonstrated that 16 batches of Cangzhu samples could be welldifferentiated and categorized into two groups, which were closely related to their species (*Atractylodes chinensis* and *A. lancea*). By loading plots of PCA and OPLS-DA, the “common peaks” **2**, **10**, and **12** were defined as “marker peaks,” which were identified as atractylodinol, (4E,6E,12E)-tetradecatriene-8,10-diyne-1,3-diyl diacetate, and atractylodin, respectively. These three “marker peaks” were then simultaneously quantified for further controlling the quality of Cangzhu, which showed acceptable linearity, both intraday and interday precisions (RSD ≤ 2.30%), repeatability (RSD ≤ 2.82%), and the recoveries of the three analytes in the range of 96.57–100.16%, with RSDs less than 1.46%. Finally, linear discriminant analysis (LDA) was successfully used to build predictive models of the group membership based on the contents of three marker peaks. Results of the present study demonstrated that HPLC-based metabolic profiling coupled with chemometric methods and quantificational determination was a very flexible, reliable, and effective way for homogeneity evaluation and quality assessment of traditional Chinese medicine.

## 1. Introduction

Chromatographic fingerprint has been approved to be an effective approach for the comprehensive quality control of traditional Chinese medicines (TCMs) and has been accepted for a long time by WHO [1, 2]. Except for this technique, metabolic profiling based on hyphenated technique, such as liquid chromatography-mass spectrometry, gas chromatography-mass spectrometry, and liquid chromatography-nuclear magnetic resonance, has showed much more powerful capabilities coupled with multivariate analysis [3]. However, the expensive instruments and highly specialized processes of a hyphenated technique do not fit well for the routine work of quality control for TCMs. Some reports have demonstrated that high-performance liquid chromatography coupled with ultraviolet detectors (HPLC-UV) based metabolic profiling was an effective strategy for the quality assessment of TCMs [2, 4]. This strategy has shown early promise to be suitable for the quality reevaluation of widely used herbal products [2]. 

Atractylodis Rhizoma, Cangzhu in Chinese, is the dried rhizome of *Atractylodes chinensis* and *A. lancea*, which belongs to the Asteraceae family and has been widely used as a traditional Chinese medicine in China [5, 6]. Cangzhu was reported in ShenNongBenCaoJing, the first Chinese pharmacopoeia written in the Han dynasty about 100–200 BC [7]. It has long been used for the treatment of rheumatic diseases, digestive disorders, night blindness, and influenza [8].* A. chinensis* and *A. lancea* have been shown to contain many effective constituents, including atractylone, hinesol, **β**-eudesmol, atractylodin, atractylodinol, and (4E,6E,12E)-tetradecatriene-8,10-diyne-1,3-diyl diacetate [7, 8]. A number of pharmacological activities of these major components were previously reported [9]. Therefore, the quality control of *A. chinensis* and *A. lancea* should be focused on the determination of the multiple active compounds. Single constituent could not be responsible for overall pharmacological activities of *A. chinensis* and *A. lancea*. However, only atrctylodin was selected as quantitative constituent for *A. chinensis* and *A. lancea* in 2010 edition of Chinese Pharmacopoeia. 

**Figure 1:**
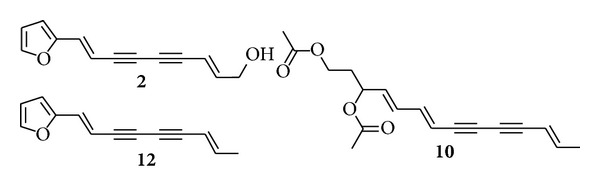
Structures of marker peaks **2** (atractylodinol), **10** ((4E,6E,12E)-tetradecatriene-8,10-diyne-1,3-diyl diacetate), and **12** (atractylodin).

Therefore, in the present study, a simple, reliable, and new high-performance liquid chromatography coupled with chemometric methods, principal component analysis (PCA), and orthogonal partial least squares discriminant analysis (OPLS-DA) was used for quality evaluation of Atractylodis Rhizoma. The newly developed method was also successfully applied to quantify the three marker compounds in 16 batches of Cangzhu samples for discriminating different species (*A. chinensis* and *A. lancea*) and acquiring quality assessment of Cangzhu (Atractylodis Rhizoma). Finally, linear discriminant analysis (LDA) was successfully used to build predictive models of the group membership based on the contents of three marker compounds. The structures of marker compounds can be seen in Figure 1.

## 2. Experimental

### 2.1. Chemicals and Reagents

Chromatography grade methanol was purchased from Merck (Darmstadt, Germany). Deionized water was purified by Milli-Q system (Millipore, Bedford, MA, USA). Samples 1–6 are collected on September 2010 as raw materials from Daxinganling district, Heilongjiang, China. Samples 7–16 are collected as decoction pieces on December 2010 from Harbin medical market. Atractylodinol (**2**) and (4E,6E,12E)-tetradecatriene-8,10-diyne-1,3-diyl diacetate (**10**) were purchased from the Research Center on Standardization of traditional Chinese medicine (Shanghai University of Chinese Medicine, China). Atractylodin (**12**) was purchased from the Chengdu JSMT Biotechnology Co. Ltd. (Chengdu, China). Their structures can be seen in Figure 1. All other reagents were of analytical grade.

### 2.2. Sample Preparation

The dried powders of *A. chinensis* or *A. lancea* (0.2 g, 60 mesh) were accurately weighed and extracted by ultrasonic with 60 kHz and 50 mL methanol solution for 1.0 hours at 40°C. Then, the resultant mixture was adjusted to the original weight and the supernatant was filtered through 0.45 *μ*m membrane before HPLC analysis.

### 2.3. HPLC Apparatus and Conditions

The analyses were performed using a Waters e2695 liquid chromatography system, equipped with a quaternary solvent delivery system, a Waters 600 controller, two Waters 600 pumps, a 2695 autosampler, a Waters 2998 photodiode array detector, and a Waters 2695 column oven. The separation was carried out on a Diamonsil C18 column (4.6 mm × 250 mm, 5 *μ*m). The gradient elution was employed using solvent A (MeOH) and solvent B (water) at 30°C; the gradient program was used as follows: initial 0–10 min, linear change from A-B (70 : 30, v/v) to A-B (75 : 25, v/v); 10–30 min, linear change to A-B (80 : 20, v/v); 30–40 min, isocratic elution for keeping A-B (80 : 20, v/v). The flow rate was set at 1.0 mL/min and the injection volume was 10 *μ*L. 

### 2.4. Standard Preparation and Calibration Curves

A methanol stock solution containing all 3 reference standards was prepared by dissolving the reference standards in methanol to final concentration of 400 *μ*g/mL for each reference standard, then the mixture stock solution was diluted to an appropriate concentration to establish calibration curves. Each calibration curve concentration was performed in triplicate. All calibration curves were constructed from peak areas of reference standards versus their concentrations. The lowest concentration of working solution was diluted with methanol to yield a series of appropriate concentrations, and the LOD and LOQ under the chromatographic conditions were separately determined at an S/N of 3 and 10, respectively. 

### 2.5. Statistical Analysis

The chromatographic data was acquired by the Waters Empower workstation for LC systems. The data could then be imported into Microsoft Excel for the addition of labels and normalization. The PCA and OPLS-DA analyses were performed using MarkerLynx software (Waters, Manchester, UK). The LDA analyss was performed using SPSS software (v.16, SPSS, USA).

## 3. Results and Discussion

### 3.1. Optimization of Sample Extraction Conditions

Since various parameters potentially affect the extraction process, the optimization of the experimental conditions is a critical step in the development of a solvent extraction method. Based on the above considerations, the extraction parameters were optimized to obtain an efficient extraction of the compounds from Atractylodis Rhizoma. In fact, extraction time (min), amount of solvent (mL), extraction temperature (°C), and ultrasonic frequency (kHz) are generally considered to be the most important factors. Optimization of the suitable extraction conditions in the Atractylodis Rhizoma extraction can be carried out by using an experimental design. In the present study, all selected factors were examined by using an orthogonal [L_9_ (3)^4^] test design (Table 1). The content of atractylodin was used as a marker to evaluate the extraction efficiency from Atractylodis Rhizoma (Anhui province). 

The results of the orthogonal test and the extreme difference analysis are presented in Table 2. The maximum extraction yield of atractylodin was 0.1519%. However, we cannot select the best extraction conditions only based on these outcomes in Table 2, and a further orthogonal analysis was warranted. Thus, the *K* and *R* values are calculated and listed in Table 2. As seen from Table 2, we can find that the influence on the mean extraction yields of the compounds decreases in the following order: C > B > D > A, according to the *R* values. So, the maximum yield of the atractylodin was obtained when extraction temperature, amount of solvent, ultrasonic frequency, and extraction time were C_2_B_2_D_3_A_2_ (40°C, 50 mL methanol, 60 kHz, and 1.0 h), respectively. According to the *R* value and the result of analysis of variance table, we can find that the extraction temperature was the most important determinant of the yield of the atractylodin. Through confirmatory test, we get the high yield of the atractylodin, with a yield (%) of 0.1552 ± 0.0013%.

### 3.2. Optimization of HPLC Conditions

**Figure 2:**
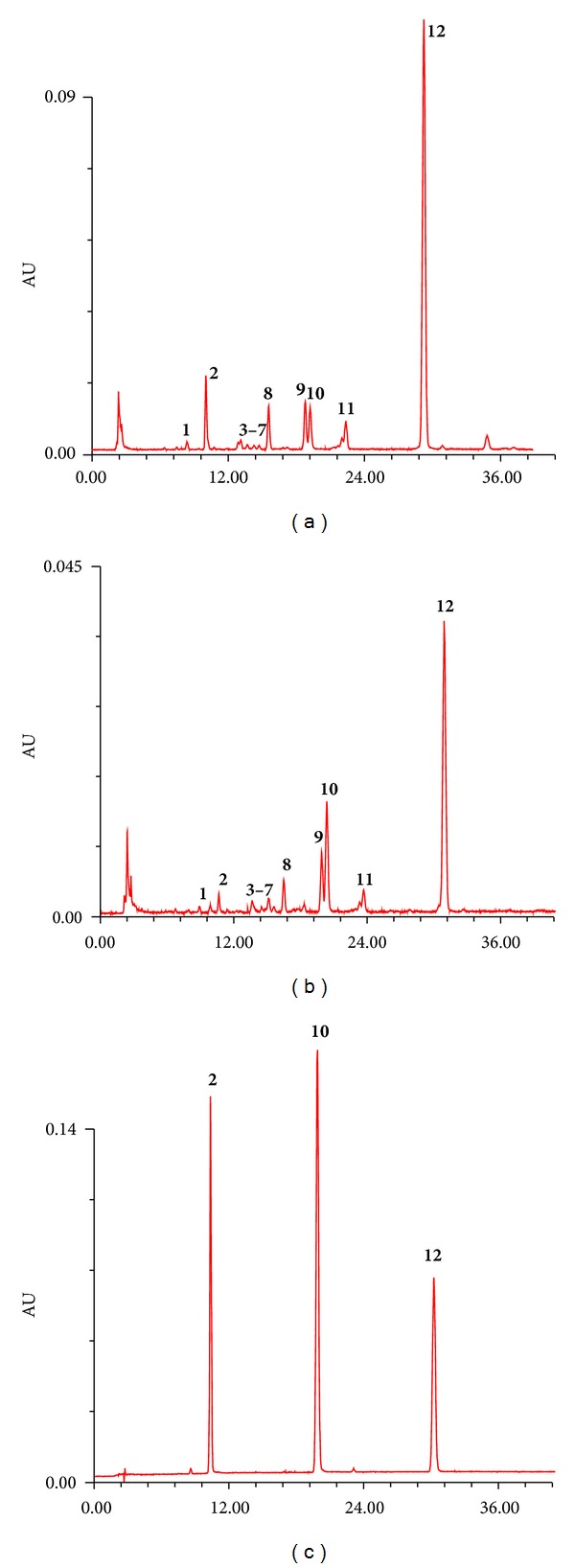
Representative HPLC chromatographic profiles at 340 nm: (a)* A. chinensis *(Neimeng province); (b) *A. lancea *(Anhui province); (c) chemical references of three marker analytes (100 *μ*g/mL for each reference standard). The retention time is defined as the minute. The structures of marker peaks **2**, **10**, and **12** can be seen in Figure 1.

Different types of chromatographic column from three manufacturers were tested (Waters symmetry C18 column, Dima Diamand C18 column, and Cosmosil 5C18-MS-II). The Atractylodis Rhizoma sample showed different retention behaviors on these columns. The analysis time did not vary significantly on three columns, while the resolution of Dima Diamand C18 column was better than the rest. There was no appreciable difference in column temperature (20–30°C) on either the peak symmetry or the resolution of the eluted peaks, but only an increase in the retention time, and so the column temperature of 30°C was used. The effect of different flow rates (0.5–1.0 mL/min) has been also examined. Tailing and poor peaks symmetry appeared at lower values of the flow rate, so 1.0 mL/min was selected as the optimum flow rate. According to 3D chromatograms, 340 nm was chosen as detection wavelength of metabolic profiling. Under these optimum conditions, all the studied chemical constituents were well separated from each other. Representative chromatograms of Atractylodis Rhizoma sample from different species (*A. chinensis* and *A. lancea*) and origins (Neimeng and Anhui provinces) and the mixed standards are shown in Figure 2. By comparison of different chromatographic profiles, the 12 common peaks were observed, which was shown in Figure 2. 

### 3.3. Quality Assessment of *A. chinensis* and *A. lancea* by PCA and OPLS-DA

16 samples of Cangzhu samples with different species (*A. chinensis and A. lancea*), origins, and processing methods were collected. The common peak data of chromatographic profiles was acquired by the Waters Empower workstation for LC systems. The data could then be imported into Microsoft Excel for the addition of labels and normalization. PCA and OPLS-DA are two sophisticated techniques widely used for reducing the dimensions of multivariate problems, which are useful tools of chemometricians for data compression and information extraction which find combinations of variables or factors that describe major trends in a data set. 

**Figure 3:**
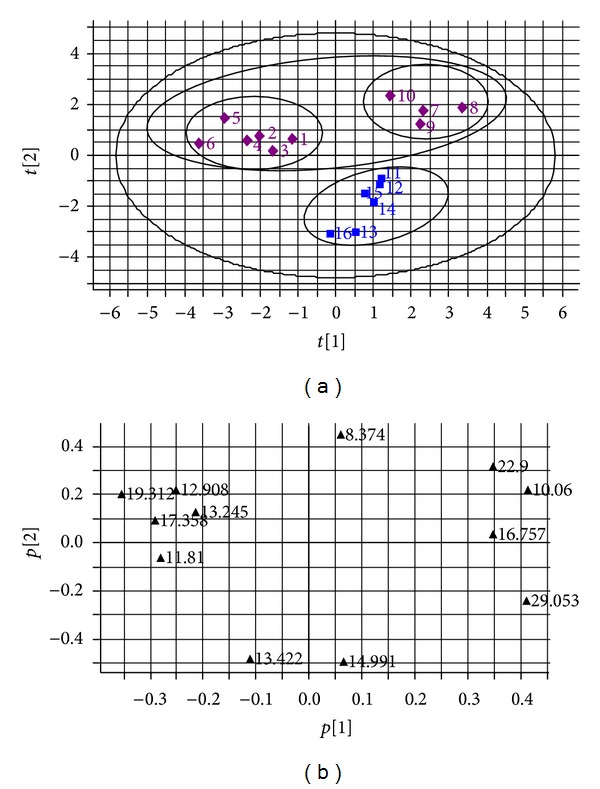
(a) Score plots from PCA; (b) loading plots from PCA, in which each variable represents the retention time (min) in chromatographic profiles.

**Figure 4:**
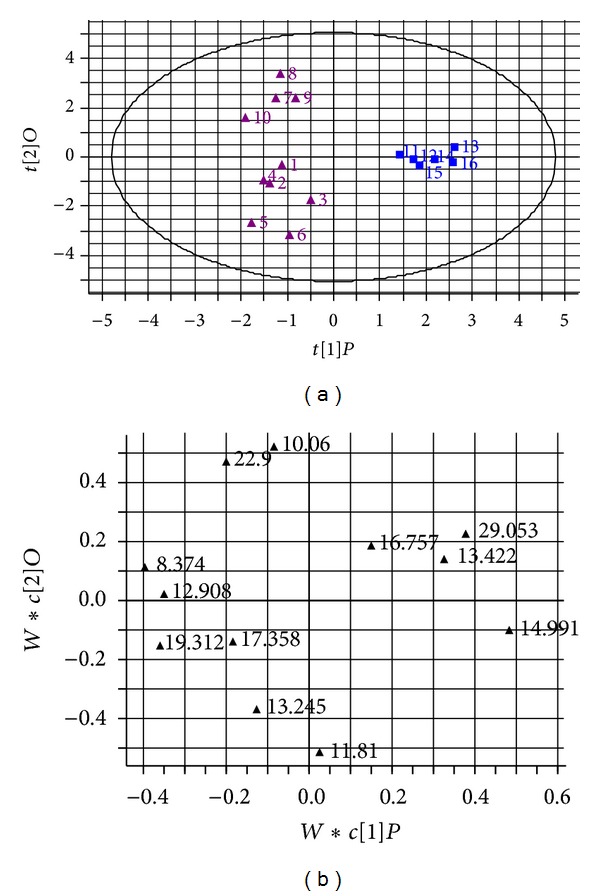
(a) Score plots from OPLS-DA; (b) loading plots from OPLS-DA, in which each variable represents the retention time (min) in chromatographic profiles.

PCA scores plot of the final combined dataset is illustrated in Figure 3(a), in which clear separation of the 16 batches of *A. chinensis* and *A. lancea *samples was observed. *R*2 (cumulative) and *Q*2 (cumulative) of the PCA model were 0.594 and 0.177, respectively. OPLS-DA was then carried out to cluster 16 batches of *A. chinensis* and *A. lancea *samples in Figure 4(a). *R*2 (cumulative) and *Q*2 (cumulative) of the OPLS-DA model were 0.936 and 0.885, respectively. Obviously, all the samples tested apparently form into two big groups according to different species. We can conclude that samples 1–10 from *A. chinensis* showed a positive correlation with *t*[2] and samples 11–16 from *A. lancea* showed a negative correlation with *t*[2] in PCA scores plot (Figure 3(a)). However, in OPLS-DA scores plot, samples 1–10 from *A. chinensis* showed a negative correlation with *t*[1] and samples 11–16 from *A. lancea* showed a positive correlation with  *t*[1] (Figure 4(a)). For further observation of samples from *A. chinensis* in PCA scores plot (Figure 3(a)), two subgroups were formed according to different origins and processing methods. Samples 1–6 are raw materials from Daxinganling districts (Heilongjiang province, China). However, samples 7–10 are decoction pieces for collecting different places of northern provinces of China.

The corresponding PCA and OPLS-DA loadings plots are illustrated in Figures 3(b) and 4(b), respectively. Clearly, three of the variables due to retention time at 8.374, 19.312, and 29.053 min play a very significant role in classifying these samples. So, these three peaks were defined as marker peaks, which were identified as atractylodinol,(4E,6E,12E)-tetradecatriene-8,10-diyne-1,3-diyl diacetate, and atractylodin, respectively, by injecting and comparing with the retention time of each standard compound, and UV spectrum was recorded using the diode array detector. Therefore, it is quite necessary to simultaneously determine these three “marker peaks” based on fingerprint profiles for further controlling the quality of *A. chinensis* and *A. lancea*.

### 3.4. Quantitative Determination of Three Marker Compounds

#### 3.4.1. Calibration Curves, Limit of Detection (LOD), and Quantification (LOQ)

Stock solutions were diluted to appropriate concentrations in order to construct calibration curves and to calculate relative response factors. The calibration curve of the individual standards was constructed using six concentrations (*n* = 3), by plotting peak areas against the concentration of analytes. The calculated results are shown in Table 3. Moreover, the regression coefficient (*R*) of three compounds was greater than 0.9996. LOD and LOQ of three compounds under the present chromatographic conditions were determined on the basis of response and slope of each regression equation at a signal-to-noise (S/N) of 3 and 10. They ranged in 2.5–200.0 *μ*g/mL. The results are summarized in Table 3.

#### 3.4.2. Precision, Repeatability, Stability, and Recovery

The quality control samples at low, medium, and high Concentrations were analyzed in a set of five on a single assay day to determine intra-day precision and were analyzed in duplicates on each of three consecutive days for inter-day variation. The values of relative standard deviations (RSDs) of the intra-day and inter-day measurement variations were all less than 2.30% for three compounds (*n* = 5).

In order to test the repeatability, six sample solutions of *A. chinensis* were prepared. RSDs of the percent contents of three marker compounds were 2.04%, 1.92%, and 2.82%, respectively. Thus, repeatability was very good. For stability test, the same sample solution was analyzed every 4 h within 3 days at room temperature. The RSD of contents of the three compounds in the same sample (Neimeng) ranged between 1.91% and 2.91%, which indicated that the sample was stable within 3 days under the experimental conditions. 

A recovery study was performed to validate the accuracy of the developed method. Sample 8 (*A. chinensis*) was spiked with different concentration levels (50, 100, and 150%) of known amounts of the compounds. The spiked samples were extracted with 10 mL ethanol following the procedure for sample preparation as described previously. The recovery was determined by comparing the amount of analyte added to the sample and the amount of analyte detected during HPLC analysis. The recoveries of the three analytes were in the range of 96.57–100.16%, with RSDs less than 1.46%. The accuracy of the proposed method was therefore found to be sufficient for the determination of the three compounds in samples from *A. chinensis* and *A. lancea*.

#### 3.4.3. Sample Analysis

A number of pharmacological activities of these marker components were previously reported [9]. However, only atractylodin was selected as quantitative constituent for *A. chinensis* and *A. lancea*, respectively, in Chinese Pharmacopoeia 2010. Qualitative and quantitative analyses of these three characteristic constituents could play an important role in evaluating and controlling quality of Atractylodis Rhizoma samples. The developed analytical method was then successfully applied to simultaneously determine the three marker components in 16 batches of Cangzhu samples obtained from different species, geographic origin, and source by HPLC method. 

The results showed that there were remarkable differences in the contents of the three compounds in the 16 batches of Atractylodis Rhizoma samples (Table 4). The contents of the three compounds in* A. chinensis* collected from different origins varied dramatically. Atractylodinol could not be detected at all in Daxinganling district of Heilongjiang province. Furthermore, the content of atractylodin in Daxinganling district of Heilongjiang province was obvious to be far less than other *A. chinensis *samples. However, the three compounds were all detected in *A. lancea*. The total content of the three compounds was up to 7.18 (mg/g) which was found in *A. lancea* of Hubei province. Averagely, the contents of atractylodin in *A. lancea* were obviously higher than those in *A. chinensis*. These variations might be on account of the different species, plant origins, harvesting time, storage conditions, and so forth. The variation in contents of marker components may cause changes in clinical efficacy. To ensure the quality of Atractylodis Rhizoma, each procedure involved should be standardized.

Finally, LDA could be used to build predictive models of the group membership based on observed characteristics of each case [1]. Here, we collected 16 Atractylodis Rhizoma as training samples to establish discriminant function of Atractylodis Rhizoma from both *A. chinensis* and *A. lancea*. Three variables were of value to the establishment of discriminant function by using stepwise method. The two discriminant functions from different species were as follows: *y*_1_ = 5.321*x*_2_ + 1.563*x*_2_ − 0.645*x*_3_ − 2.039 and *y*_2_ = −13.107*x*_1_ + 0.911*x*_2_ + 1.267*x*_3_ − 3.649, where *y*_1_ denotes samples attributed to *A. chinensis* and *y*_2_ denotes samples attributed to* A. lancea*. These three variables (*x*_1_–*x*_3_) denoted the contents of atractylodinol, (4E,6E,12E)-tetradecatriene-8,10-diyne-1,3-diyl diacetate, and atractylodin, respectively.

**Figure 5:**
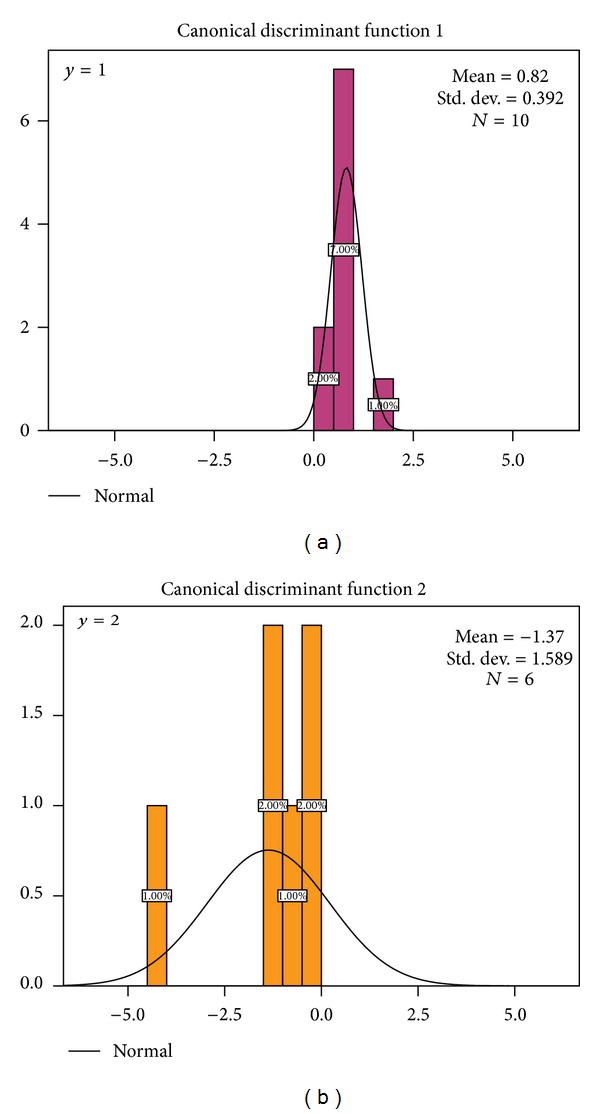
The plots of linear discriminant analysis. (a) Normal distribution of ten *A.chinensis* samples; (b) normal distribution of six *A. lancea*. samples.

As a result, training samples from Atractylodis Rhizoma could be classified into two groups, which was consistent with that of PCA and OPLS-DA. Figures 5(a) and 5(b) are representative of a normal distribution of ten *A. chinensis* and six *A. lancea *samples, respectively. According to discriminant functions *y*_1_ and *y*_2_, 100% and 93.8% of trained samples were correctly classified to the *A. chinensis* and *A. lancea*, respectively. It is possible that there is one sample from Hubei which is obviously different from other *A. lancea* samples. So, a 6.2% misjudgment was present in the discriminant function *y*_2_.

## 4. Conclusions 

Our current study indicates that HPLC-based metabolic profiling coupled with chemometric methods is a valuable tool for the authentication of botanical species of Cangzhu and can also be useful for quality control of Atractylodis Rhizoma. Therefore, it can be concluded that this newly developed method is very flexible, reliable, and effective for homogeneity evaluation and quality assessment of traditional Chinese medicine.

## Figures and Tables

**Table 1:** Orthogonal experimental design factors and levels for ultrasonic-assisted extraction process.

Variables	Levels		
	1	2	3
(A) Extraction time (min)	30	60	90
(B) Amount of solvent (mL)	40	50	60
(C) Extraction temperature (°C)	30	40	50
(D) Ultrasonic frequency (kHz)	40	50	60

**Table 2:** Orthogonal experimental design test results of the ultrasonic-assisted extraction process.

No.	A	B	C	D	Atractylodin (%)
1	1	1	1	1	0.1077
2	1	2	2	2	0.1519
3	1	3	3	3	0.1328
4	2	1	2	3	0.1418
5	2	2	3	1	0.1485
6	2	3	1	2	0.1205
7	3	1	3	2	0.1330
8	3	2	1	3	0.1365
9	3	3	2	1	0.1363
*K*_1_	0.1308	0.1275	0.1216	0.1308	
*K*_2_	0.1369	0.1456	0.1433	0.1351	
*K*_3_	0.1353	0.1299	0.1381	0.1370	
*R *	0.0061	0.0181	0.0217	0.0062	

Note: *K*_*i*_ is obtained by adding any number of columns corresponding to *i* factor. *R* is the difference between the maximum value and the minimum value of *K*_*i*_ of any columns.

**Table 3:** Calibration parameters of HPLC analysis for the 3 compounds. The structures of marker peaks of **2**, **10**, and **12** can be seen in Figure 1.

No.	Regression equation	Linear range (*μ*g/mL)	*R *	LODs (*μ*g/mL)	LOQs (*μ*g/mL)
**2**	*y* = 114567*x* − 49750	2.5–200.0	0.9996	0.1	0.3
**10**	*y* = 38856*x* − 16893	2.5–200.0	0.9997	0.1	0.3
**12**	*y* = 114052*x* − 142322	2.5–200.0	0.9999	0.1	0.3

**Table 4:** The measurement results of marker compounds in *A. chinensis* and *A. lancea* (mg/g). The structures of **2**, **10**, and **12** can be seen in Figure 1.

No.	Source	Geographical regions	**2**	**10**	**12**
1	*A. chinensis *	Jianan, Daxinganling, Heilongjiang	—	0.948	0.050
2	*A. chinensis *	Huma, Daxinganling, Heilongjiang	—	1.382	0.044
3	*A. chinensis *	Wuchagou, Daxinganling, Heilongjiang	—	1.378	0.089
4	*A. chinensis *	Wogonglu, Daxinganling, Heilongjiang	—	1.239	0.037
5	*A. chinensis *	Daxinganling, Heilongjiang	—	1.401	0.022
6	*A. chinensis *	Liaoning	—	0.958	0.095
7	*A. chinensis *	Harbin, Heilongjiang	0.227	2.621	3.065
8	*A. chinensis *	Neimeng	0.451	1.108	4.757
9	*A. chinensis *	Neimeng	0.570	4.655	5.488
10	*A. chinensis *	Neimeng	0.143	3.318	2.142
11	*A. lancea *	Anhui	0.048	1.380	1.552
12	*A. lancea *	Anhui	0.087	1.357	2.297
13	*A. lancea *	Hubei	0.014	4.049	7.165
14	*A. lancea *	Jiangsu	0.091	1.369	3.456
15	*A. lancea *	Shanxi	0.074	2.485	3.067
16	*A. lancea *	Shandong	0.059	4.167	3.675

## References

[1] Xia YG, Yang BY, Wang QH (2009). Quantitative analysis and chromatographic fingerprinting for the quality evaluation of *Forsythia suspensa* extract by HPLC coupled with photodiode array detector. *Journal of Separation Science*.

[2] Wang Z, Hu H, Chen F (2012). Metabolic profiling assisted quality assessment of *Rhodiola rosea* extracts by high-performance liquid chromatography. *Planta Medica*.

[3] Liu E-H, Qi L-W, Li K, Chu C, Li P (2010). Recent advances in quality control of traditional Chinese medicines. *Combinatorial Chemistry and High Throughput Screening*.

[4] Lan K, Zhang Y, Yang J, Xu L (2010). Simple quality assessment approach for herbal extracts using high performance liquid chromatography-UV based metabolomics platform. *Journal of Chromatography A*.

[5] http://www.tropicos.org/NameSearch.aspx.

[6] State Pharmacopoeia Committee (2010). *Pharmacopoeia of People’s Republic of China (2010 version)*.

[7] Guo F-Q, Huang L-F, Zhou S-Y, Zhang T-M, Liang Y-Z (2006). Comparison of the volatile compounds of Atractylodes medicinal plants by headspace solid-phase microextraction-gas chromatography-mass spectrometry. *Analytica Chimica Acta*.

[8] Meng H, Li G-Y, Dai R-H (2011). Two new polyacetylenic compounds from *Atractylodes chinensis* (DC.) Koidz. *Journal of Asian Natural Products Research*.

[9] Resch M, Heilmann J, Steigel A, Bauer R (2001). Further phenols and polyacetylenes from the rhizomes of *Atractylodes lancea* and their anti-inflammatory activity. *Planta Medica*.

